# *Myo5b* knockout mice as a model of microvillus inclusion disease

**DOI:** 10.1038/srep12312

**Published:** 2015-07-23

**Authors:** Fernando Cartón-García, Arend W. Overeem, Rocio Nieto, Sarah Bazzocco, Higinio Dopeso, Irati Macaya, Josipa Bilic, Stefania Landolfi, Javier Hernandez-Losa, Simo Schwartz, Santiago Ramon y Cajal, Sven C. D. van Ijzendoorn, Diego Arango

**Affiliations:** 1 Group of Molecular Oncology, CIBBIM-Nanomedicine, Vall d’Hebron University Hospital Research Institute (VHIR), Universitat Autònoma de Barcelona, Barcelona, Spain; 2 CIBER de Bioingeniería, Biomateriales y Nanomedicina (CIBER-BBN), Zaragoza, Spain; 3 Department of Cell Biology, University of Groningen, University Medical Center Groningen, Groningen, The Netherlands; 4 Department of Pathology, Vall d’Hebron Hospital, Barcelona, Spain; 5 Group of Drug Delivery and Targeting, CIBBIM-Nanomedicine, Vall d’Hebron University Hospital Research Institute (VHIR), Universitat Autònoma de Barcelona, Barcelona, Spain

## Abstract

Inherited *MYO5B* mutations have recently been associated with microvillus inclusion disease (MVID), an autosomal recessive syndrome characterized by intractable, life-threatening, watery diarrhea appearing shortly after birth. Characterization of the molecular mechanisms underlying this disease and development of novel therapeutic approaches is hampered by the lack of animal models. In this study we describe the phenotype of a novel mouse model with targeted inactivation of *Myo5b*. *Myo5b* knockout mice show perinatal mortality, diarrhea and the characteristic mislocalization of apical and basolateral plasma membrane markers in enterocytes. Moreover, in transmission electron preparations, we observed microvillus atrophy and the presence of microvillus inclusion bodies. Importantly, *Myo5b* knockout embryos at day 20 of gestation already display all these structural defects, indicating that they are tissue autonomous rather than secondary to environmental cues, such as the long-term absence of nutrients in the intestine. *Myo5b* knockout mice closely resemble the phenotype of MVID patients and constitute a useful model to further investigate the underlying molecular mechanism of this disease and to preclinically assess the efficacy of novel therapeutic approaches.

Microvillus inclusion disease (MVID) is an autosomal recessive syndrome affecting the intestinal epithelium^1^,^2^. It was first described in 1978 and it is characterized by the onset of abundant neonatal watery diarrhea that most commonly starts within the first days of life, and can cause the loss of up to 30% of body weight within 24 h^1^,^2^. In some cases (<20%), MVID manifests at later times, within the first 3–4 months of life.

The diagnosis of MVID is based on the detection of morphological abnormalities in the intestinal epithelium using a combination of light and electron microscopy. Histological examination of the small intestinal mucosa reveals a variable degree of villus atrophy. In addition, there is a characteristic accumulation of periodic acid–Schiff (PAS)-positive cytoplasmic granules in intestinal epithelial cells^3^,^4^. Transmission electron microscopy (TEM) of intestinal epithelial biopsies is used to confirm the diagnosis. Ultrastructural defects in small intestinal enterocytes include the shortening of microvilli and the presence of distinctive cytoplasmic vacuoles lined by microvilli, known as microvillus inclusion bodies^5^.

Inactivating mutations in *MYO5B* have recently been associated with the majority of cases of MVID^6^,^7^. *MYO5B* codes for the unconventional myosin Vb, an actin-based motor involved in plasma membrane recycling through its interactions with RAB GTPases^8^. The loss of a functional Myosin Vb protein results in profound protein trafficking defects in enterocytes leading to the mistargeting of apical and basolateral proteins^9^,^10^. These abnormalities in the structure of the apical brush border and the mislocalization of membrane proteins are likely responsible for the absorption defects and the watery diarrhea observed in these patients, but the detailed molecular mechanisms remain to be fully elucidated. Currently, the only treatment options available for this uniformly fatal disease are total parenteral nutrition and intestinal transplant^11^.

Here we describe the phenotype of the first animal model with targeted inactivation of *Myo5b*. Mice deficient for this myosin show perinatal mortality, watery diarrhea and the characteristic structural defects of patients with MVID. This study provides formal demonstration of *MYO5B* mutations as the cause of microvillus inclusion disease. Moreover, the availability of this mouse model will decisively contribute to shed new light on the underlying molecular mechanisms of this disease and the development and testing of new therapeutic approaches for MVID patients.

## Results

### Survival of *Myo5b* knockout mice

**Figure 1:**
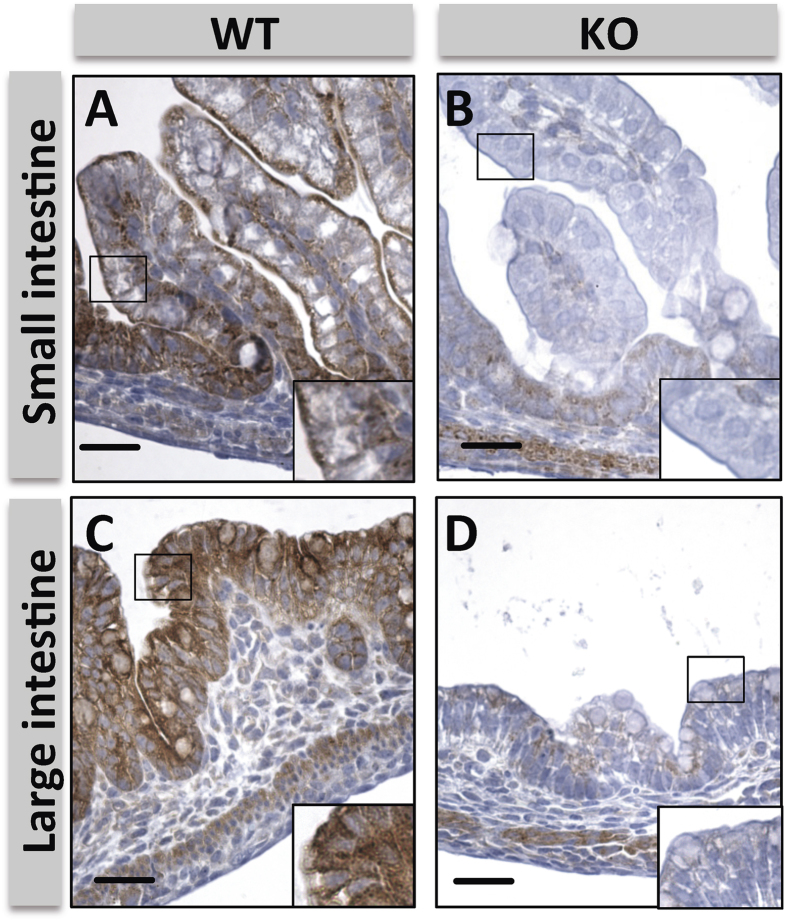
Effects of *Myo5b* inactivation. Immunostaining showing Myosin Vb levels in the small (**A,B**) and large (**C,D**) intestine of *Myo5b* wild type (**A** and **C**) and knockout (**B** and **D**) newborn mice. Scale bar: 50 μm.

**Figure 2:**
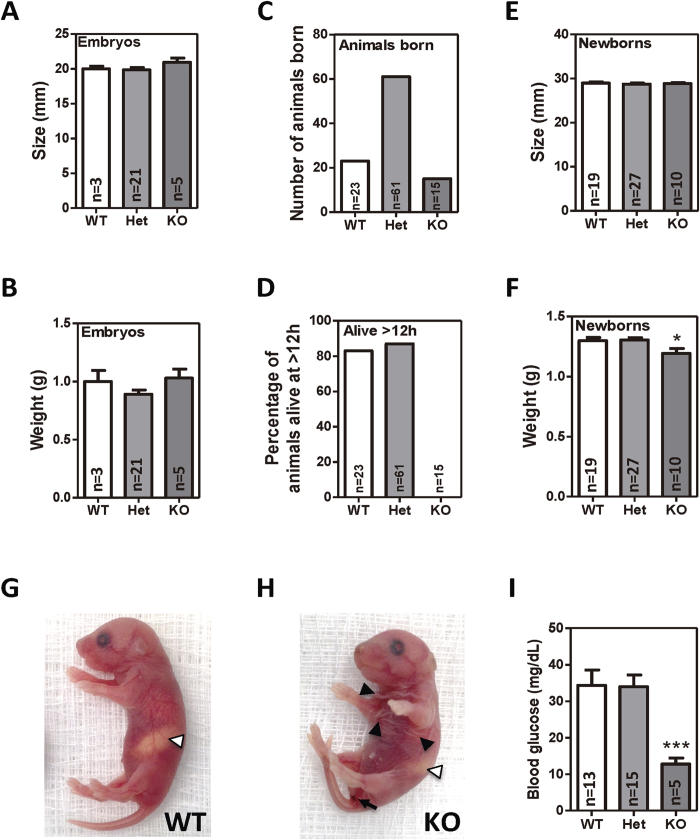
Phenotype of *Myo5b* E20 embryos and newborn mice. Size (**A**) and weight (**B**) of E20 embryos by *Myo5b* genotype. The mean ± SEM is shown. N = number of animals per group. (**C**) Genotype of 99 mice born from crossing heterozygous male and female mice. (**D**) Percentage of these 99 mice that were alive 12 h after birth. Size (**E**) and weight (**F**) of newborn mice by *Myo5b* genotype (mean ± SEM). Newborn wild type (**G**) and *Myo5b* knockout (**H**) mice showing the presence of the milk spot (white arrowhead), wrinkled skin (black arrowhead) and evidence of diarrhea (arrow). (**I**) Histogram showing average (±SEM) blood glucose levels in *Myo5b* wild type, heterozygous and newborn mice. *p < 0.05; ***p < 0.001 (Student’s T-test).

Homologous recombination was used to introduce a targeting cassette including the mouse En2 splice acceptor and the SV40 polyadenylation sequences after exon 4 of *Myo5b*, which is predicted to generate a null allele through splicing to a lacZ “gene trap” element (Supplementary Figure 1)^12^. As expected, mice homozygous for the trapped allele showed no Myosin Vb expression in their intestine (henceforth referred to as *Myo5b* knockout mice; Fig. 1). Wild type, heterozygous or *Myo5b* knockout embryos at day 20 of gestation (E20) showed no difference in their size or weight (Fig. 2A,B). Animals were born at Mendelian ratios (n = 99; Chi-square test, p = 0.14; Fig. 2C), but *Myo5b* knockout mice invariably died within the first 12 h after being born (Fig. 2D). No differences were observed between wild type and knockout newborn mice in their body size (Fig. 2E) or the gross histology of the gastrointestinal tract (Supplementary Figure 2) or other organs studied, including the lungs, liver, central nervous system, heart, pancreas and spleen. No cyanotic episodes or respiratory distress was observed in *Myo5b* knockout mice. However, newborn *Myo5b* knockout mice showed reduced bodyweight compared to wild type and heterozygous mice (Fig. 2F). Moreover, knockout mice showed signs of diarrhea and wrinkled skin, possibly due to dehydration (Fig. 2G,H). Although newborn *Myo5b* knockout mice showed no suckling defects (presence of a milk spot; Fig. 2G,H), they had significantly reduced blood glucose levels compared to wild type and heterozygous littermates (Fig. 2I). This is consistent with the watery diarrhea and absorption defects observed in patients with MVID^1^,^2^ and likely contributed to the death of the *Myo5b* knockout mice within hours of birth^13^.

### Mislocalization of apical brush border proteins in the enterocytes of *Myo5b* knockout mice

**Figure 3:**
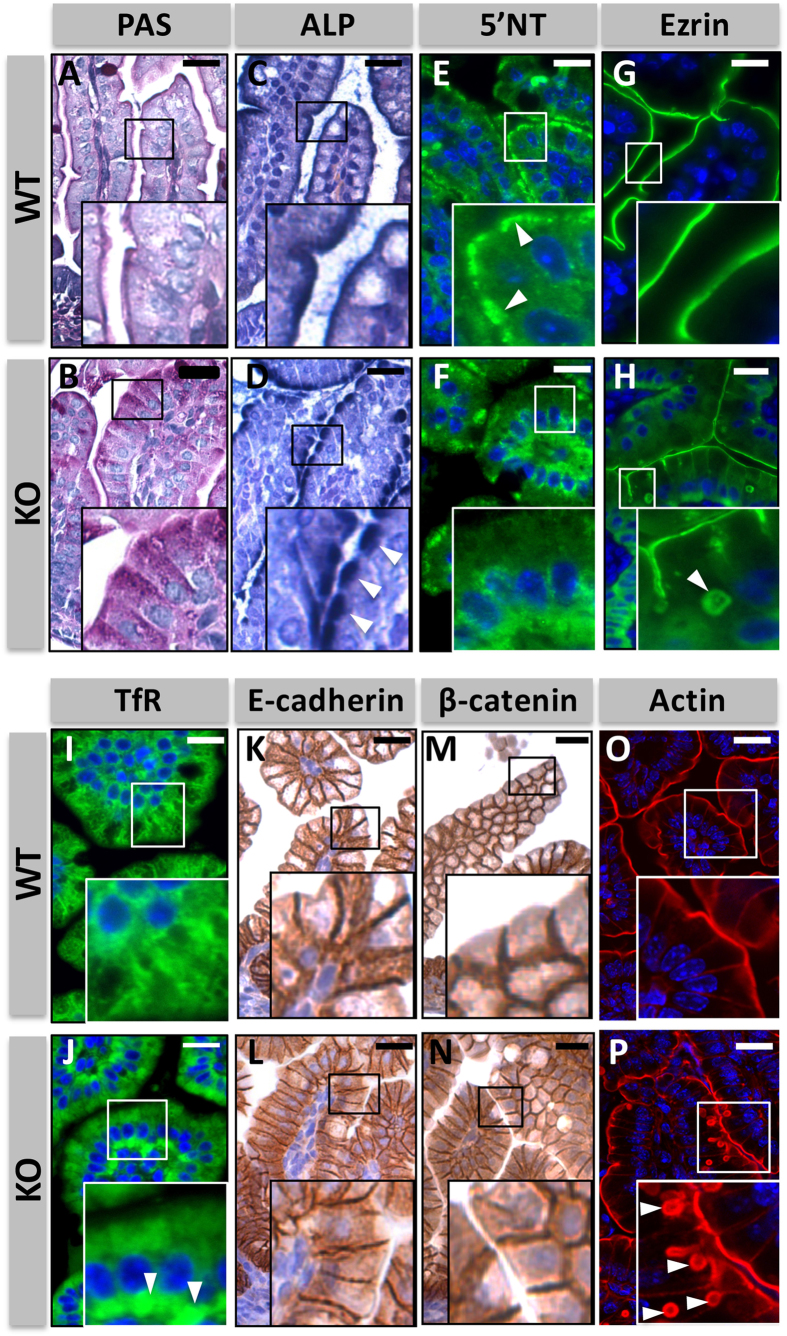
Changes in the localization of apical and basolateral protein markers in Myo5b knockout newborn mice. Periodic acid–Schiff (PAS) staining (**A,B**), alkaline phosphatase (ALP) staining (**C,D**; arrowheads indicate the subapical accumulation of ALP in *Myo5b* knockout mice), immunostaining of 5’-Nucleotidase (5’NT; **E,F**; arrowheads indicate the apical distribution of 5’NT in wild type mice), ezrin (**G,H**; arrowhead indicates an intracellular ezrin-coated vesicle in *Myo5b* knockout mice), transferrin receptor (TfR; **I,J**; arrowheads indicate the basal accumulation of TfR in *Myo5b* knockout mice), E-Cadherin (**K,L**), β-catenin (**M,N**) and actin (**O,P**; arrowheads indicate intracellular actin-coated vesicles in *Myo5b* knockout mice) in *Myo5b* wild type and knockout newborn mice. Scale bar: 25 μm.

At the ultrastructural level, the intestinal enterocytes of *Myo5b* knockout newborn mice showed the characteristic cytoplasmic accumulation of periodic-acid Schiff (PAS) staining observed in MVID patients^3^,^4^ (Fig. 3A,B). Moreover, proteins normally expressed in the apical membrane of intestinal enterocytes such as alkaline phosphatase (ALP; Fig. 3C,D), 5’-Nucleotidase (5’NT; Fig. 3E,F) and ezrin (Fig. 3G,H) mislocalized to the basolateral membrane or the cytoplasm. Transferrin receptor (TfR) accumulated in the basal cytoplasm of enterocytes from *Myo5b* knockout mice (Fig. 3I,J), while other basolateral markers such as E-cadherin (Fig. 3K,L) and β-catenin (Fig. 3M,N) were unaffected. Notably, in some epithelial cells of *Myo5b* knockout mice ezrin (Fig. 3G,H) and actin (Fig. 3O,P) were found in circular cytoplasmic structures, closely resembling microvillus inclusions. These findings are in good agreement with the protein sorting defects observed in the intestinal epithelium of patients with MVID^3^,^6^,^14^,^15^. Importantly, these structural defects were also observed in E20 embryos (Supplementary Figure 3A–L).

### Ultrastructural defects in the brush border of the enterocytes of *Myo5b* knockout mice

**Figure 4:**
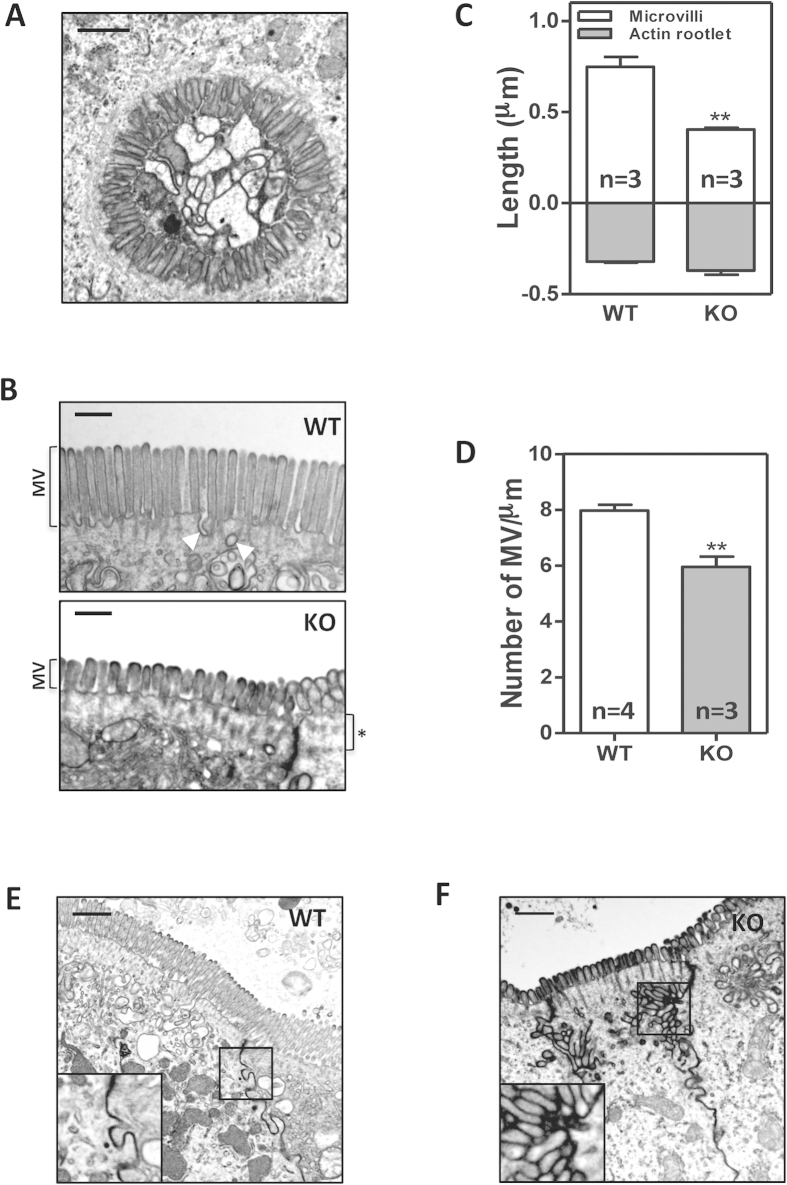
Ultrastructural defects in the intestinal epithelium of knockout *Myo5b* E20 embryos. (**A**) TEM micrograph showing microvillus inclusion bodies in *Myo5b* knockout E20 embryos (scale bar 0.5 μm). (**B**) Apical microvilli of enterocytes in *Myo5b* wild type and knockout E20 embryos (scale bar 0.5 μm). MV: microvilli; White arrowheads: subapical microvesicles; asterisk indicates a subapical region devoid of microvesicles. (**C**) Average (±SEM) length of microvilli projecting into the lumen and actin rootlets. (**D**) Number of microvilli observed per micrometer in transverse sections of the brush border. The normal junction between enterocytes in wild type *Myo5b* E20 embryos is shown in (**E**). Scale bar 1 μm. Microvilli-like structures could be observed in the lateral membrane of enterocytes in *Myo5b* knockout mice (**F**). Scale bar 1 μm. **p < 0.01 (Student’s T-test).

**Figure 5:**
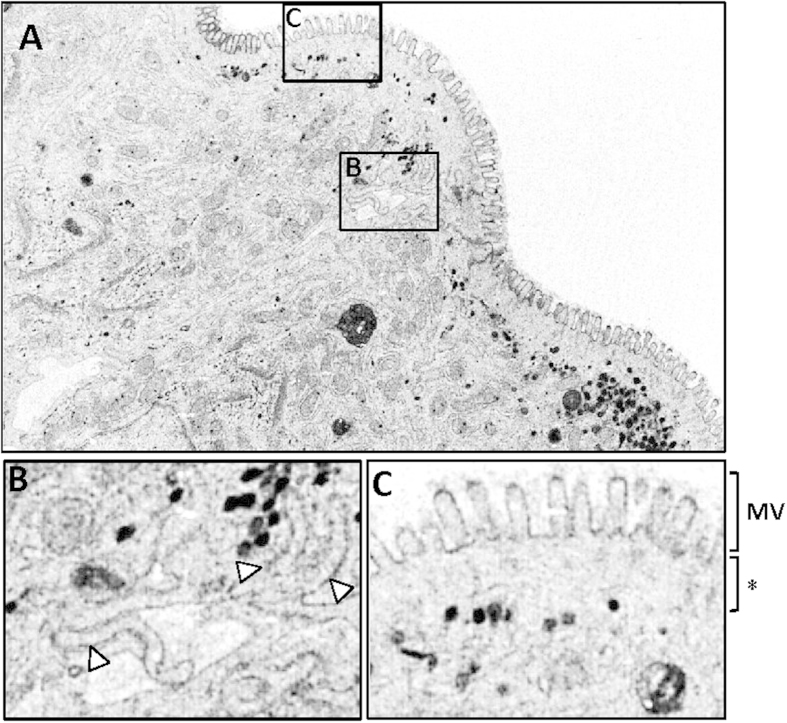
Ultrastructural defects in the intestinal epithelium of MVID patients. (**A**) Transmission electron microscopy pictures from a duodenum biopsy of a MVID patient carrying a homozygous *MYO5B* nonsense mutation (c.4366C > T, p.1456X). (**B**) Larger magnification of a region containing lateral microvilli-like structures (white arrowheads). (**C**) Detail of the apical region containing short/poorly packed microvilli (MV) and a subapical area devoid of microvesicles (asterisk).

Transmission electron microscopy (TEM) analysis of the intestinal epithelium revealed the presence of microvillus inclusion bodies in the cytoplasm of absorptive cells from *Myo5b* knockout E20 embryos (Fig. 4A). In addition, the apical surface of the intestinal enterocytes showed widespread microvilli atrophy and reduced packing with areas with few/absent microvilli (Fig. 4B–D and Supplementary Figure 4) and presence of microvilli in the lateral plasma membrane (Fig. 4E,F). Vesicles were frequently observed at the apical plasma membrane in *Myo5b* wild type mice but not in the *Myo5b* knockout animals, where a characteristic accumulation of vesicles could be observed underneath the terminal web (Fig. 4B and Supplementary Figure 4). These ultrastructural abnormalities closely resemble the phenotype observed in the intestinal epithelium of patients with MVID (Fig. 5A–C)^4^,^5^.

## Discussion

We describe here the phenotype of the first mouse model with targeted inactivation of *Myo5b*. Germline mutations in this gene are associated with microvillus inclusion disease (MVID)^6^, a congenital disorder of the intestinal epithelium causing persistent life-threatening watery diarrhea^1^,^2^. Myosin Vb is an actin-based molecular motor with a key role in vesicle trafficking and plasma membrane recycling through its interaction with the small GTPases RAB11 and RAB8^8^,^9^. It is not surprising, therefore, that inactivation of either *Rab11a* or *Rab8a* in the mouse intestine resulted in nutrient malabsorption, intracellular accumulation of apical proteins in intestinal epithelial cells, shortening of microvilli and microvillus inclusion bodies^16^,^17^. Interestingly, inactivation of the small GTPase *Cdc42* also caused microvilli shortening and microvillus inclusions in intestinal epithelial cells^18^. However, diarrhea, one of the hallmarks of MVID patients, was not observed in *Rab11* or *Cdc42* knockout mice^17^,^18^, and *Rab8* knock out mice survived for approximately 5 weeks after birth, more closely resembling the phenotype of late onset MVID. Importantly, no mutations in *RAB8* or *RAB11* GTPases have been identified in *MYO5B* mutation negative MVID patients^19^, suggesting that *Myo5b* deficient mice represent the optimal animal model for human microvillus inclusion disease.

Indeed, *Myo5b* knockout mice showed all the typical features observed in patients with early onset MVID, the most common form of this disease accounting for >80% of the cases^4^. *Myo5b* deficient mice showed no overt defects during embryonic development, having normal size and weight. However, newborn *Myo5b* knockout mice showed watery diarrhea and died during the first 12 h of life, likely due to dehydration and/or reduced nutrient availability secondary to absorption defects, as exemplified by the low blood glucose levels observed, although the contribution of each of these symptoms to the death of Myo5b deficient mice cannot be conclusively determined. The body weight reduction observed in *Myo5b* knockout mice (8% in approximately 6 h) is consistent with the fluid loss reported in early onset MVID patients (>30% of body weight in 24 h; i.e., 7.5% in 6 h)^3^. In previous studies, the perinatal mortality of *Klf4* or *Scd2* newborn knockout mice was attributed to a 5–10% reduction of body weight due to transepidermal water loss^20^,^21^. In humans, this rapid rate of dehydration would result in hypovolemic shock leading to death, as observed in *Myo5b* knockout mice. On the other hand, newborn mice have previously been shown to go through a transitory phase of severe hypoglycemia (about 10 mg/dL within 2 h of birth) until glucose levels are restored due to gluconeogenesis and eventually nutrient absorption of maternal milk^13^,^22^,^23^. Consistent with the phenotype observed in *Myo5b* knockout animals, the incapacity of newborn mice to overcome the postnatal hypoglycemia has been shown to be fatal within 18 h^22^. Moreover, although no reproducible defects have been reported in other organs of MVID patients and no histological abnormalities were observed in the *Myo5b* knockout mice, additional studies of the function of other organs and the possible contribution to the death of Myo5b deficient mice are warranted.

Consistent with the changes observed in the intestinal epithelium of MVID patients^1^,^2^, important structural defects were observed in the enterocytes of *Myo5b* knockout newborn mice, including the mislocalization of apical and basolateral markers, microvillus atrophy and the presence of microvillus inclusion bodies. Collectively, this study provides formal demonstration of the inactivation of *Myo5b* as the cause of microvillus inclusion disease. Moreover, these results indicate that the absence of a functional Myosin Vb protein, rather than the presence of pathogenic Myosin Vb mutations^24^, is responsible for the intestinal defects observed in MVID patients. In addition, the presence of ultrastructural defects in the enterocytes of Myo5b-deficient E20 embryos indicates that this phenotype is tissue-autonomous. However, the characteristic villus atrophy observed in patients with MVID was not observed in *Myo5b* knockout E20 embryos or newborn mice (Supplementary Figure 2E), suggesting that this phenotype is secondary to environmental cues, such as the prolonged absence of nutrients in their gastrointestinal tract^25^. The *Myo5b* knockout model described here will be instrumental for the characterization of the molecular mechanisms downstream of Myosin Vb responsible for the phenotype observed in patients with MVID, and should significantly contribute to the identification of novel therapeutic approaches for these patients.

It has been reported that up to 75% of MVID patients die before 9 months of age^4^. Different pharmacological approaches have been used in an attempt to stop/reduce the severe diarrhea in these patients, but none of them has proven effective^4^. Patients are dependent on total parenteral nutrition, which over time often causes liver damage and sepsis. Small-bowel transplantation is the only option available to avoid parenteral nutrition and improve the quality of life and the long-term prognosis of these children^11^. However, intestinal transplantation is associated with high rates of rejection and/or mortality^11^, and additional therapeutic options are urgently needed for these patients. The *Myo5b* knockout model described here will constitute an ideal system to preclinically test the efficiency of possible new treatment options, including pharmacological or gene therapy using for example autologous reimplantation of intestinal epithelium grown *ex vivo* following restoration of functional Myosin Vb^26^.

In conclusion, we describe here the phenotype of *Myo5b* knockout mice, which closely phenocopies human early-onset microvillus inclusion disease. These experiments confirm the important role of Myosin Vb in the formation of the apical brush border and the sorting of apical and basolateral proteins in intestinal absorptive cells, and formally demonstrate that the loss of a functional Myosin Vb protein is responsible for the phenotype observed in MVID patients. The availability of this mouse model of MVID will not only contribute to the characterization of the molecular pathological mechanisms downstream of Myosin Vb leading to novel therapeutic approaches, but also provides an ideal system to preclinically test different treatment options.

## Methods

### Generation of *Myo5b* knockout mice

*Myo5b*^*tm1a(KOMP)Wtsi*^ targeted ES cells (C57BL/6N, agouti) were obtained from the KOMP repository at UC Davis^12^,^27^. After expansion, cells were injected into donor blastocysts and transplanted into pseudopregnant females. Chimeric male offspring were mated to C57BL/6N females to confirm germ line transmission. Animals were genotyped by PCR. The primers used were: Myo5b-F: 5’-CCA GTT CCT TGG GGA CAT AA-3’, loxP-F: 5’-GAG ATG GCG CAA CGC AAT TAA TG-3’ and Myo5b-R: 5’-AGT GAT GCT GTC CTG AGT GTA CTG G-3’. The initial *tm1a* allele generates a null allele through splicing to a lacZ trapping element, including the mouse En2 splice acceptor and the SV40 polyadenylation sequences (Supplementary Figure 1). Heterozygous *Myo5b*^*tm1a(KOMP)Wtsi*^ mice were intercrossed to obtain animals homozygous for the targeted *Myo5b* allele (knockout mice). All animal experiments were carried out according to procedures approved by the Ethics Committee for Animal Experimentation at Vall d´Hebron Research Institute.

### Transmission electron microscopy

Duodenal samples were collected from *Myo5b* wild type and *Myo5b* knockout E20 embryos (at least 3 animals per genotype). Samples were fixed with 2.5% glutaraldehyde and 2% paraformaldehyde and processed following standard procedures. Ultra-thin sections were mounted on copper grids, contrasted with uranyle acetate/lead citrate double-staining, and observed in a Jeol JEM-1400 (Jeol LTD, Tokyo, Japan) transmission electron microscope equipped with a Gatan Ultrascan ES1000 CCD camera. The brush border architecture was evaluated on a minimum of 12 enterocytes per animal. Microvilli length (actin rootlet and actin core bundles) and microvilli density (microvilli/μm) were measured using ImageJ software. Duodenum biopsy sample from a MVID patient carrying a homozygous *MYO5B* nonsense mutation (c.4366C > T, p.1456X) was obtained after removal of the diseased intestine during the transplantation procedure^10^. The sample was fixed in 2% glutaraldehyde in phosphate buffer, rinsed in 6.8% sucrose in phosphate buffer, and postfixed in a solution of 1% osmium tetroxide in 0.1 mol/L sodium cacodylate buffer containing 11.2% potassium ferrocyanide. Samples were dehydrated with ethanol and processed according to standard procedures upon embedding. Ultra-thin sections were mounted on copper grids and contrasted with uranyle acetate and lead citrate double-staining.

### Histology and immunohistochemistry

*Myo5b* wild type and knockout embryos were obtained at day 20 of gestation (E20) and sacrificed by decapitation on ice-cold PBS. Newborns were collected within 6 hours of birth, and sacrificed by decapitation. Both embryos and newborn mice were weighted and measured using a caliper. Blood samples were obtained form tail clips of newborn mice. Glucose levels were measured with a Glucocard G+ meter (Menarini diagnostics, Barcelona). The small and large intestine were dissected from embryos or newborn mice, their length measured and then fixed overnight with 4% formalin, dehydrated by serial immersion in 50%, 70%, 96%, 100% ethanol and xylene and embedded in paraffin. In parallel, formalin-fixed duodenal samples obtained from newborns were cryoprotected in 15% sucrose in PBS overnight, then 30% sucrose overnight. Samples were then immersed in OCT (VWR) and frozen on dry-ice for cryosectioning.

For MYO5B, E-cadherin and β-catenin immunostaining, the NovoLink polymer detection system (Novocastra Laboratories) was used. Inmunostaining was carried out in 3 μm tissue sections, after deparaffination and antigen retrieval with 10 mM citrate buffer pH 6.0 in a pressure cooker for 4 min. The antibodies used were: anti-MYO5B (Atlas antibodies HPA040902; 1:800); anti-E-cadherin (BD Bioscience cat# 610181; 1:100) and β-catenin (BD Bioscience cat# 610154; 1:100). For Ezrin and Transferrin receptor inmunostaining, epitopes were retrieved at 100 °C for 20 minutes in 10 mM citric acid, 0.05% Tween 20 pH6.0. For 5’-nucleotidase, epitopes were retrieved with 10 mM Tris Base, 1 mM EDTA Solution, 0.05% Tween 20, pH 9.0. Non-specific binding sites were blocked with 5% FCS and 1% BSA in PBS overnight. Primary antibodies were diluted in blocking solution with 0.05% Tween 20 at 37 °C for 2 hours followed by 1 hour incubation with Alexa-Fluor-488-conjugated (Ezrin and 5’-nucleotidase) or Alexa-Fluor-543-conjugated secondary antibody (Transferrin receptor). Primary antibodies used were: anti-Ezrin (Tebu Bio, 1:100), anti-Transferrin receptor (Invitrogen, 1:100), anti-5’-nucleotidase (Abgent, 1:50). Nuclei were stained with DAPI and slides were mounted with DAKO mounting medium. For alkaline phosphatase activity detection, slides were incubated with staining solution for a maximum of 1 h at 37 °C. Then, counterstained with hematoxylin and washed with distillated water before mounting. Staining solution contains 0.4 mg/mL 5-Bromo-4-chloro-3-indolyl phosphate p-toluidine (Sigma), 0.5 mg/mL of nitro blue tetrazolium (Sigma), 100 mM MgCl_2_ (Sigma), 2 mM Levamisole hydrochloride (Santa cruz Biotechnology), 5 mM Sodium azide (Sigma) and 0.15 mM of 1-methoxy-5-methylphenazinium methyl sulphate in 100 mM Tris pH 9.5 (Sigma). Periodic acid-Schiff staining was performed after deparaffination. Briefly, the slides were immersed in 0.5% periodic acid solution (Sigma) for 5 min, washed in distillated water and placed in Schiff reagent (Sigma) for 15 min. Then counterstain with hematoxylin and mounted. For F-Actin staining, 10 μm thick duodenal cryosections were stained with rhodamine phalloidin (Cytoeskeleton), nuclei were counterstained with DAPI and slides were mounted with Prolong antifade reagent (Invitrogen). Fluorescence microscopy pictures were taken with a confocal microscope (FV1000 Olympus).

## Additional Information

**How to cite this article**: Cartón-García, F. *et al.* *Myo5b* knockout mice as a model of microvillus inclusion disease. *Sci. Rep.* **5**, 12312; doi: 10.1038/srep12312 (2015).

## Supplementary Material

Supplementary Information
