# Supplementary material for: Myo5b knockout mice as a model of microvillus inclusion disease
Source: Sci Rep. 2015 Jul 23;5:12312. doi: 10.1038/srep12312 (PMC4511872; doi:10.1038/srep12312)
Supplement: Supplementary Information [file srep12312-s1.pdf]

Myo5b knockout mice as a model of microvillus inclusion disease

Fernando Cartón-García[1,2], Arend Overeem[3], Rocio Nieto[1,2], Sarah Bazzocco[1,2], Higinio Dopeso[1,2], Irati Macaya[1,2], Josipa Bilic[1,2], Stefania Landolfi[4], Javier Hernandez-Losa[4], Simo Schwartz Jr[5], Santiago Ramon y Cajal[4], Sven C. D. van Ijzendoorn[3] and Diego Arango[1,2]

[1]Group of Molecular Oncology, CIBBIM-Nanomedicine, Vall d'Hebron University Hospital, Research Institute (VHIR), Universitat Autònoma de Barcelona, Barcelona, Spain; [2]CIBER de Bioingeniería, Biomateriales y Nanomedicina (CIBER-BBN); Zaragoza, Spain; [3] Department of Cell Biology, University of Groningen, University Medical Center Groningen, Groningen, The Netherlands; [4]Department of Pathology, Vall d'Hebron Hospital, Barcelona, Spain; [5]Group of Drug Delivery and Targeting, CIBBIM-Nanomedicine, Vall d'Hebron University Hospital, Research Institute (VHIR), Universitat Autònoma de Barcelona, Barcelona, Spain.

Supplementary Figure 1

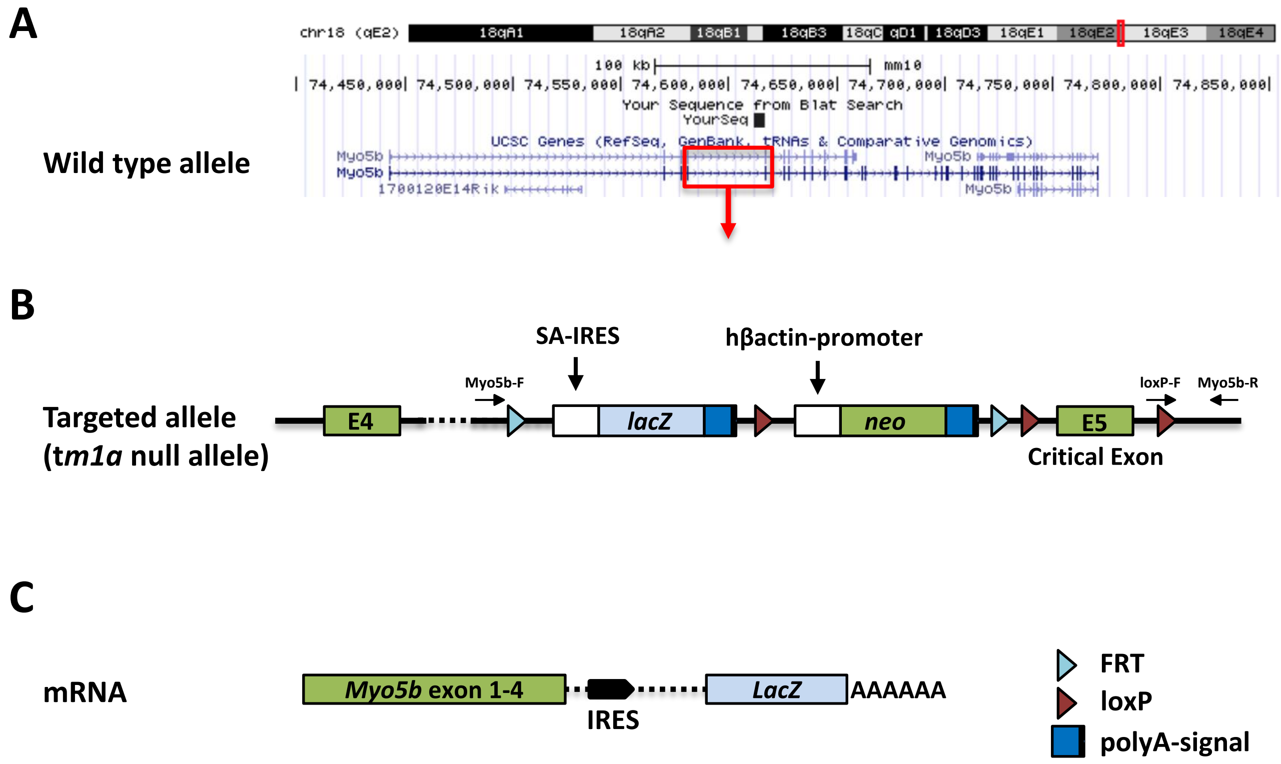

Supplementary Figure 1: Targeted inactivation of *Myo5b*. The wild type *Myo5b* murine allele (**A**) and the targeted allele (**B**) are shown. Using homologous recombination, a trapping element was introduced in intron 4-5 of *Myo5b*, including the mouse *En2* splice acceptor and the SV40 polyadenylation sequences, as well as a neomycin resistance cassette under the control of the human β-actin promoter, flanked by a FRT sites. This allele is predicted to result in a truncated mRNA containing the first 4 exons of *Myo5b* followed by an IRES sequence and the *LacZ* coding sequence (**C**). The strategy used has the potential to be converted into a conditional knockout system by crossing these mice with an Flp deleter mouse (not pursued here). Horizontal black arrows in (**B**) indicate the location of the PCR primers used for genotyping.

Supplementary Figure 2

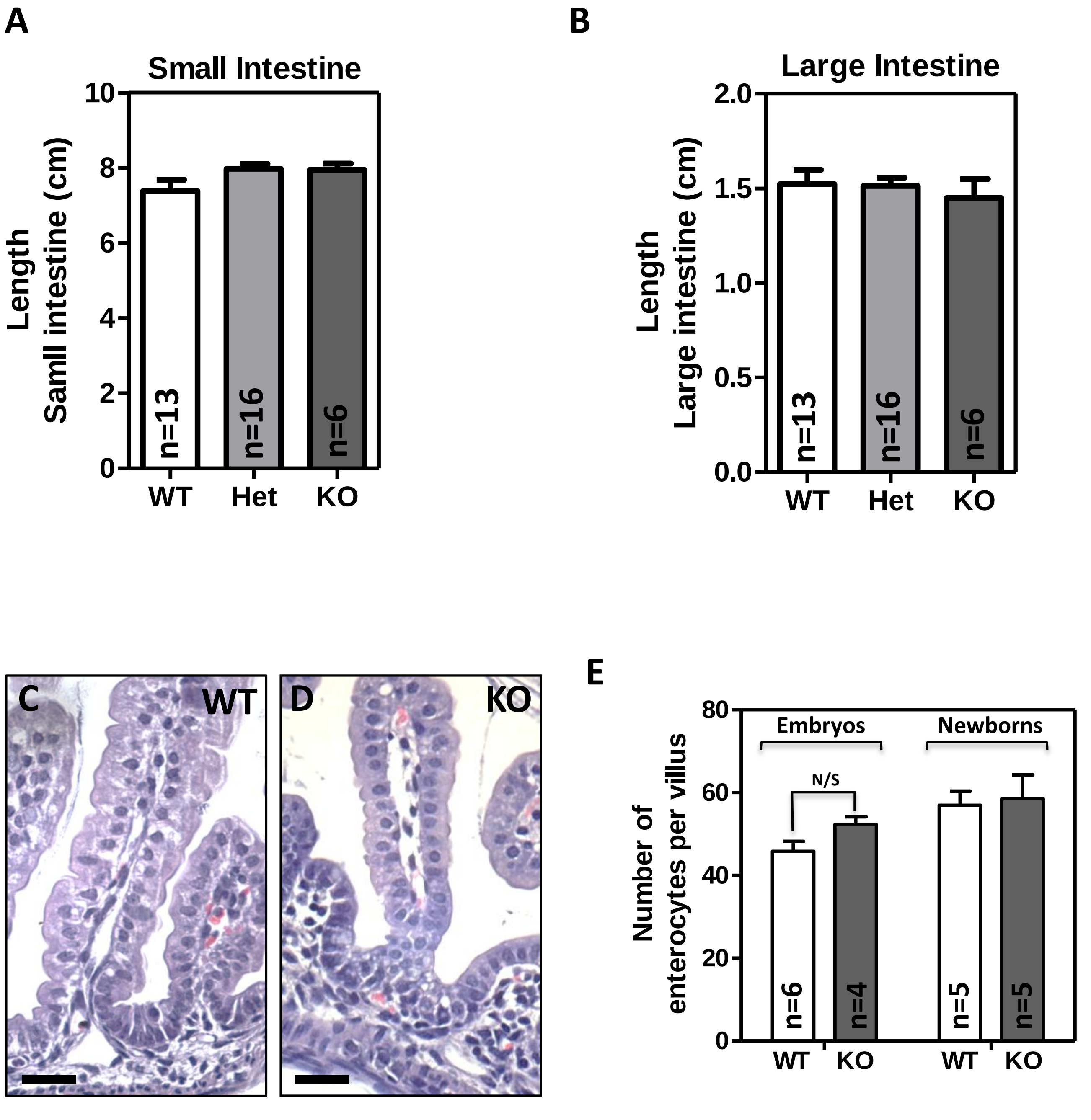

Supplementary Figure 2: Histology and anatomy of the intestine of *Myo5b* knockout mice. (**A-B**) Average length (±SE) of the small (**A**) and large (**B**) intestine of newborn mice that are wild type, heterozygous or knockout for *Myo5b*. N: number of animals per group. (**C-D**) Sections from formalin-fixed, paraffin-embedded samples from the small intestine of newborn *Myo5b* wild type (**C**) and knockout (**D**) mice were stained with hematoxylin and eosin. Scale bar 25µm. (**E**) The number of epithelial cells in at least five longitudinally sectioned villi per mouse was scored blinded from the sample identity in E20 embryos and newborn mice. The average (±SE) is shown. N: number of animals per group.

Supplementary Figure 3

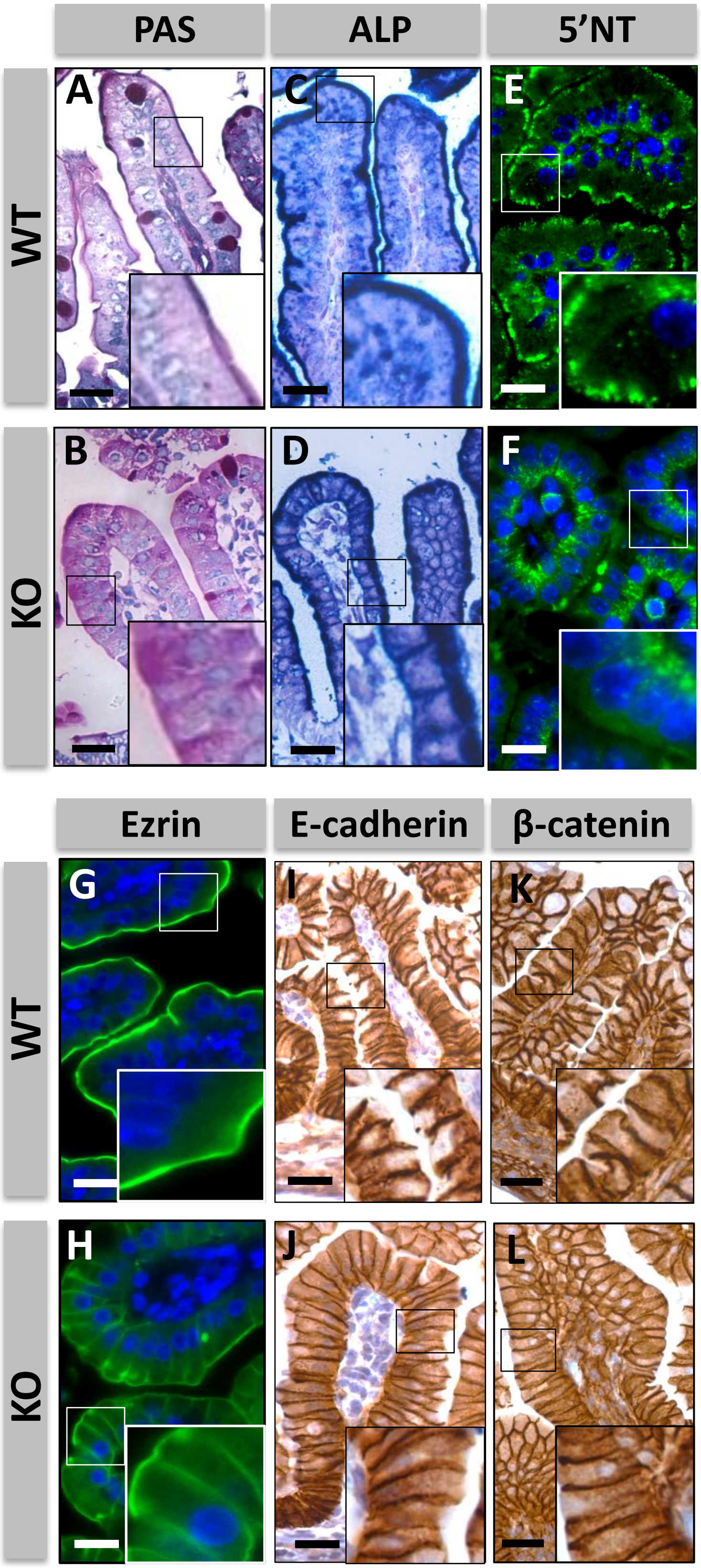

Supplementary Figure 3: Structural changes in the intestinal epithelial cells of *Myo5b* E20 embryos. (**A-L**) Periodic acid–Schiff (PAS) staining (**A-B**), alkaline phosphatase staining (ALP; **C-D**), immunostaining of 5'-Nucleotidase (5'NT; **E-F**), ezrin (**G-H**), E-Cadherin (**I-J**) and β-catenin (**K-L**) in *Myo5b* wild type and knockout E20 embryos. Scale bar: 25 μm.

Supplementary Figure 4

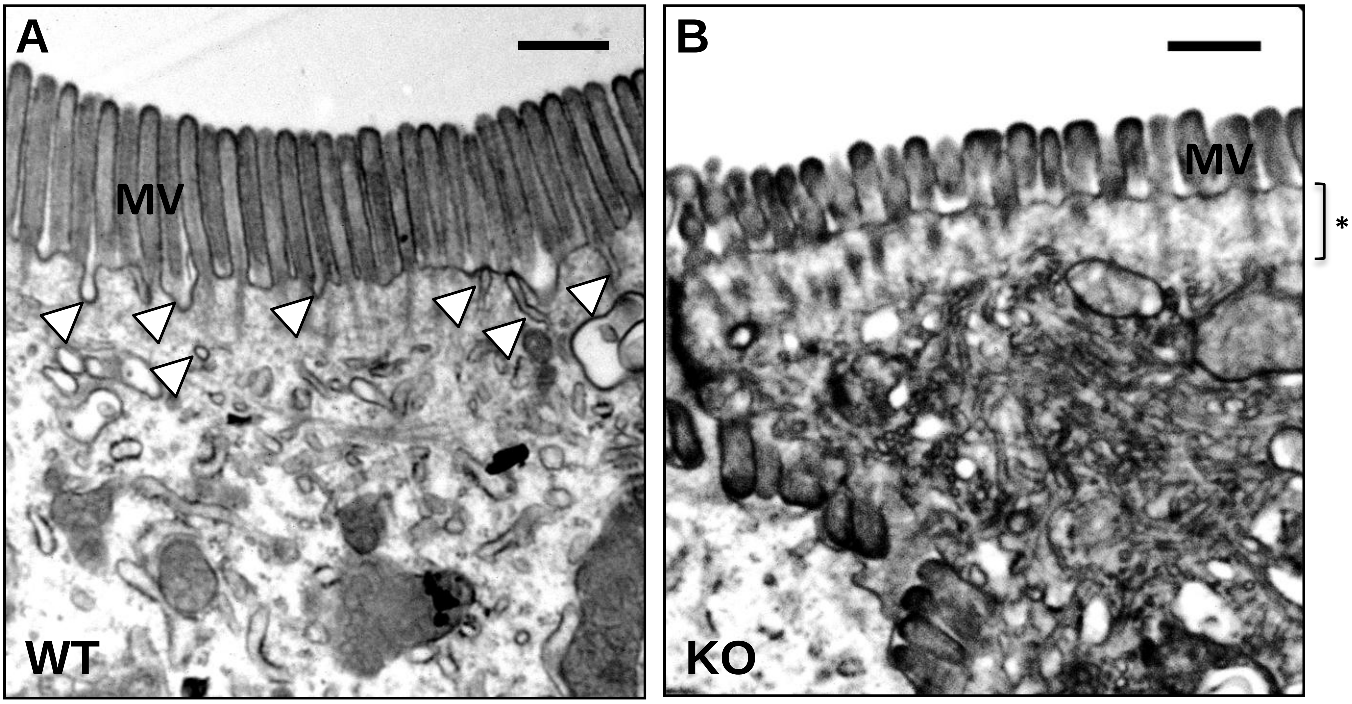

Supplementary Figure 4: Apical region of enterocytes from *Myo5b* wild type and knockout mice. Representative transmission electron microscopy micrographs of the apical region of enterocytes from *Myo5b* wild type (**A**) and knockout (**B**) mice. Enterocytes from *Myo5b* knockout mice showed shorter microvilli (MV) and a subapical region lacking microvesicle trafficking (asterisk). White arrowheads indicate apical vesicle trafficking in the *Myo5b* wild type enterocytes. Scale bar: 0.5 µm.
